# Improvement in RNA quantity and quality in cervico-vaginal cytology

**DOI:** 10.1186/s12985-020-1282-x

**Published:** 2020-01-20

**Authors:** Gun Oh. Chong, Hyung Soo Han, Seon Duk Lee, Yoon Hee Lee

**Affiliations:** 1 Department of Obstetrics and Gynecology, School of Medicine, Kyungpook National University, Daegu, Republic of Korea; 2 Department of Obstetrics and Gynecology, Kyungpook National University Chilgok Hospital, Daegu, Republic of Korea; 3 Molecular Diagnostics and Imaging Center, School of Medicine, Kyungpook National University, Daegu, Republic of Korea; 4 Department of Physiology, School of Medicine, Kyungpook National University, Daegu, Republic of Korea

**Keywords:** Cervico-vaginal cytology, mRNA, Quantity, Quality

## Abstract

The separation of exfoliated cells from the brushes used during cervico-vaginal smears is difficult, a problem which may affect the quality of ribonucleic acid (RNA) extracted. We compared the results of RNA extraction from cervico-vaginal cytology samples according to the type of tubes, preservative solutions, and storage temperature. The samples included exfoliated cervico-vaginal cytological specimens from patients with human papilloma virus 16, positive for cervical intraepithelial neoplasia or cervical cancer. Exfoliated cells were obtained by shaking a brush in a conventional rigid vial tube or squeezing the brush in a soft vial tube. RNA quantity and quality were compared between the two tubes. The concentration and purity of RNA (A260/A280 and A260/A230 ratios) was compared amongst five groups: Group 1, standard frozen storage; Group 2–4, RNA stabilization reagents with room temperature [RNAlater RNA Stabilization Reagent, RNAprotect cell Reagent and AllProtect Tissue Reagent]; and Group 5, Surepath Preservative fluid. To demonstrate the utility of the extracted RNA for PCR-based cDNA synthesis, GAPDH and E6 were targeted and gel band densities of GAPDH and E6 were measured. The median RNA concentration was significantly higher in the soft tubes compared with the rigid tubes (100.2 vs. 7.1 ng/μL, *p* = 0.0209). The purity of the RNA was higher in soft vial tubes than in rigid vials, as measured by A260/280 and A260/230 ratios. The RNA concentration, purity, and GAPDH density of groups 1, 2 and 3 were significantly higher than those of groups 4 and 5. Moreover, E6 density of group 1 and 2 was significantly higher than that of group 3, 4 and 5. The use of soft tubes enhanced the mRNA quantity and quality in cervico-vaginal cytology. The products of mRNA extraction using RNAlater RNA Stabilization Reagent and RNAprotect Cell Reagent at room temperature were comparable to those obtained by conventional frozen storage. Our protocol improved the yield and quality of RNA and might produce better results for molecular analysis in cervico-vaginal cytology.

## Main text

Exfoliated cytology has been proposed as a method which can provide information about epithelial cells such as screening for precancerous lesions or RNA expression [1]. Cytology using exfoliated cells is non-invasive, painless, convenient, and low cost, and can be used to obtain mRNA from cervico-vaginal cells for functional genomics studies.

Previous studies have demonstrated the important role of long non-coding RNAs, circular RNAs, microRNAs and mRNAs in cervical cancer development and progression [2–4]. High-quality RNA is a pre-requisite for gene expression studies. RNA is highly labile and degrades quickly if stored under improper conditions. RNA molecules are susceptible to a variety of secondary metabolites, and enzymatic degradation by ribonucleases (RNases), causing problems during extraction [5].

The separation of exfoliated cells from the brush is important for RNA extraction. Because most RNA stabilization reagents are sticky, and the tube is rigid, separation of the exfoliated cells from the brush is difficult. Traditionally, RNA is stored at − 20 °C, − 80 °C or in liquid nitrogen to provide protection from degradative reactions. However, shipping and storage of frozen RNA is expensive, requires special handling and Ultra-Low-Temperature freezers, and is time sensitive. The extraction of DNA and RNA from liquid-based cytology specimens has previously been studied [6, 7].

To the best of our knowledge, comparison of mRNA extraction methods for molecular analysis of cervico-vaginal cytology samples has not been reported. Therefore, the aim of this study was 1) to compare methods of RNA extraction of cervico-vaginal cytology as they relate to the type of tubes, preservative solutions, and storage temperature and 2) to establish the best technique for molecular tests such as real-time reverse transcriptase reaction (qRT PCR) or RNA-seq at room temperature.

**Fig. 1:**
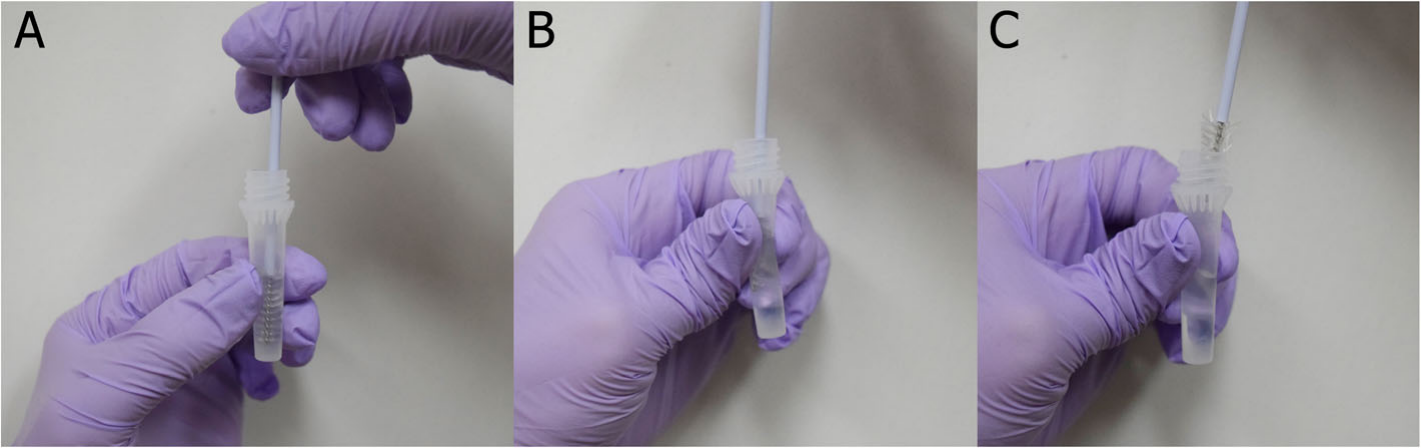
Separation of exfoliated cervico-vaginal cells from brush using elastic and soft tubes (**a**) insertion of the brush into the soft tube (**b**) squeezing of the tube and brush (**c**) removal of the brush

After obtaining approval from the Institutional Review Board of our institution, and informed written consent from the subjects, cervico-vaginal smears were collected using pap brush-lines (Bion, Guri, Korea) in patients with HPV 16-positive cervical intraepithelial neoplasia or cervical cancer. The brush was placed into the cervix and rotated 360 degrees in a clockwise direction. The samples were transported in DNase, RNase and pyrogenic free tubes. To obtain the cells from the cytobrush, the cytobrush was shaken in rigid tube, or the tube and brush were squeezed in a soft tube (Fig. 1). Detailed steps to separate exfoliated cervico-vaginal cells from the brush using soft elastic tubes were as follows: First, the brush was inserted into the soft tube (Fig. 1a). Second, the upper portion of the soft tube and cytobrush were grasped by the thumb and index finger (Fig. 1b). Third, the cytobrush was removed while maintaining this grasp, and the soft tube and cytobrush were squeezed one time (Fig. 1c). Samples were stored at − 20 °C without preservative solution for 7 days. RNA was extracted from each patient’s swab brush samples using Qiagen RNAeasy Plus Mini Kits (Qiagen, Hilden, Germany) according to the manufacturer’s protocol. The quality of the isolated RNA was measured using a Nano One spectrophotometer (ThermoFisher Scientific, Waltham, USA), set to measure absorbances at 230, 260, and 280 nm. The 260/280 and 260/230 absorbance ratios were calculated to evaluate RNA purity. The 260/280 values between 1.8 and 2.0 and 260/230 values between 1.8 and 2.2 suggest the RNA was free of contamination [8]. RNA quantity and quality were compared between conventional rigid tubes (Wuxi NEST Biotechnology Co., China) and soft tubes (Soft Dropper Tube, Biosciences, Inc. Korea) from four patients and a total of 24 samples (6 samples per patient).

To compare the conventional frozen RNA extraction method and a room-temperature RNA extraction method using RNA stabilization reagents, we divided 11 patients’ cervico-vaginal swabs, obtained under strict conditions to avoid contamination, into five groups. We obtained five cervico-vaginal samples per patient and a total of 55 samples were analyzed. A soft elastic tube was used to separate exfoliated cells from the cervico-vaginal cytology samples as previous described methods. The protocol for Group 1 was to keep the swab brush in an empty tube, and that for Group 2 was to keep the swab brush samples in the RNAlater RNA Stabilization Reagent (Qiagen, Hilden, Germany). Samples from Group 3 were kept in RNAprotect cell Reagent (Qiagen, Hilden, Germany) and those from Group 4 were stored in the AllProtect Tissue Reagent (Qiagen, Hilden, Germany). Samples from Group 5 were kept in BD SurePath™ Preservative Fluid (BD TriPath, Burlington NC, USA). Samples from Groups 1 and 5 were stored at − 20 °C for 7 days, and those from Groups 2, 3, and 4 were stored at room temperature for 7 days. We compared the RNA concentration and quality of each of the five types of samples for each patient using previously described methods. cDNA was synthesized from mRNA using TOPscript™ cDNA Synthesis Kits (Ensynomics, Daejeon, Korea) according to the manufacturer’s protocol.

Reverse Transcription Polymerase Chain Reaction (RT PCR) was performed to compare the expression of human housekeeping genes and human Papillomavirus E6 gene in each sample. Glyceraldehyde 3-phosphate dehydrogenase (GAPDH) primers were used to detect human housekeeping genes, and E6 primers were used to detect HPV E6 genes. Primer sequences are described in Additional file 1: Table S1.

PCR temperature cycling for GAPDH was as follows: pre-heating for 5 min at 95 °C, followed by 33 cycles of 30 s at 95 °C, 30 s at 58 °C, 25 s at 72 °C, and a final extension of 5 min at 72 °C. PCR conditions for the E6 region of the HPV primer set were as follows: pre-heating for 5 min at 95 °C, followed by 33 cycles of 30 s at 95 °C, 30 s at 57 °C, 30 s at 72 °C, and a final extension of 5 min at 72 °C. After confirming the GAPDH PCR results, E6 PCR was performed by selecting 6 samples with high GAPDH expression levels. All PCR amplifications were performed with appropriate positive and negative controls. The PCR products of the GAPDH and E6 were separated by electrophoresis on 1.8% agarose gels. The electrophoresis images of PCR products were analyzed and quantified using ImageJ, an open source image processing program designed for the analysis of scientific images.

Statistical analysis was performed using SPSS version 21.0 software (SPSS, Chicago, IL, USA). Differences between subsets were evaluated using Student’s t-tests and Mann-Whitney tests, and differences between proportions were compared using chi-squared tests. ANOVA was used to compare the mean of each value among the groups and *post-hoc* tests were performed using Student-Newman-Keuls. Spearman’s correlation analysis was used to clarify the relationships between gene expression and RNA quantity and quality. A *p* value of < 0.05 was considered statistically significant.

**Fig. 2:**
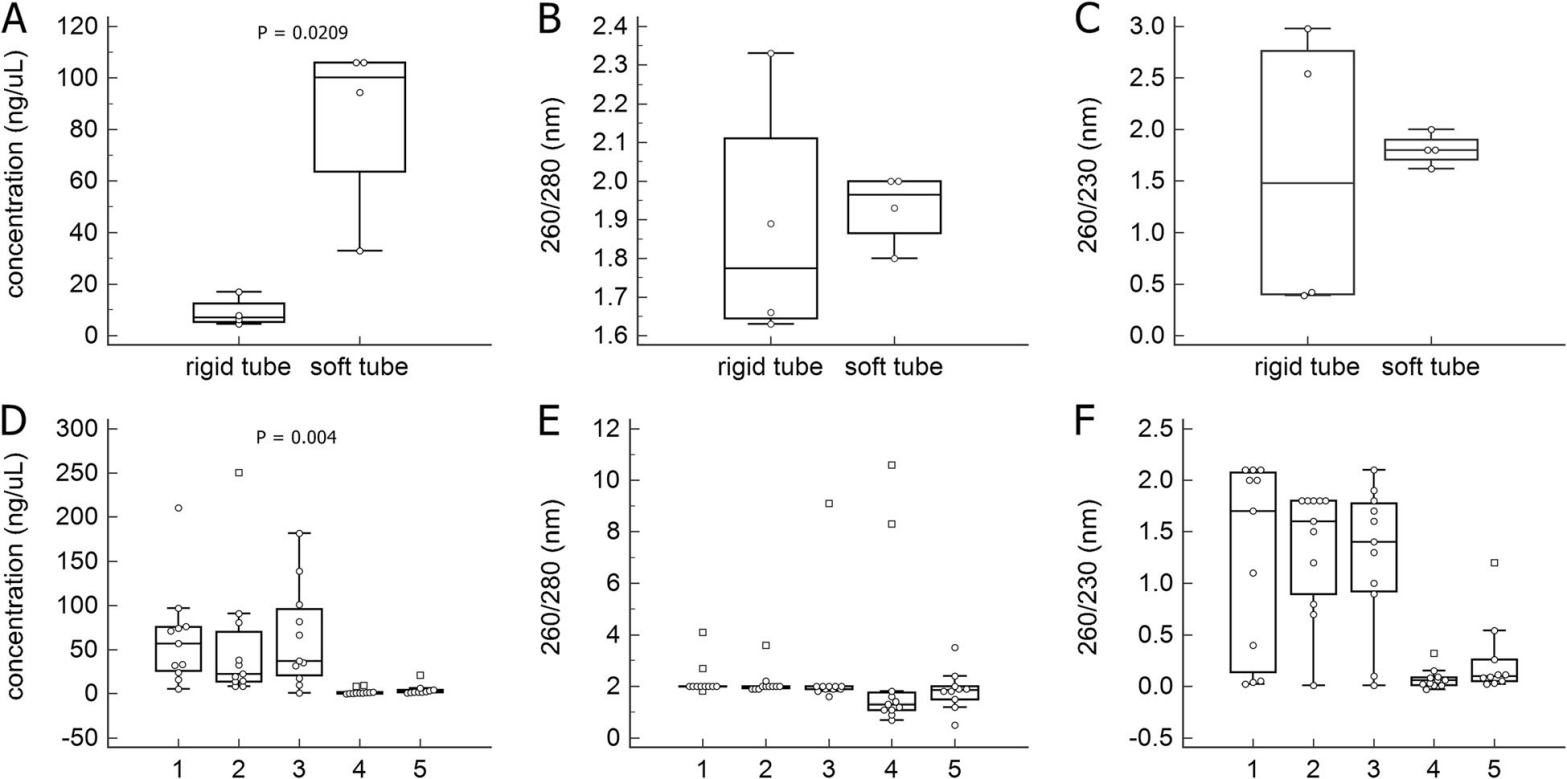
Comparisons of RNA concentration (**a**), 260/280 ratio (**b**) and 260/230 ratio (**c**) between rigid and soft tubes. Comparisons of RNA concentration (**d**), 260/280 ratio (**e**) and 260/230 ration (**f**) among the five groups

The median RNA concentration was significantly higher in soft tubes (100.2 ng/μL, range 33.0–105.9 ng/μL) than in rigid tubes (7.1 ng/μL, range 4.5–17.0 ng/μL; *p* = 0.0209). 260/280 ratios of between 1.8 and 2.0 were observed for all four samples in soft tubes, but for only one sample in rigid tubes. 260/230 ratios of between 1.8 and 2.2 were observed for three samples in soft tubes, but for none in rigid tubes (Table 1, Fig. 2a-c).

**Table 1:** Comparison of RNA quantity and quality between rigid and soft tubes

No	Concentration (ng/μL)			260/280		260/230	
	Rigid tube	Soft tube	*P* value	Rigid tube	Soft tube	Rigid tube	Soft tube
1	6.2	105.9		1.63	2.00	0.39	1.80
2	17.0	105.9		1.89	2.00	0.42	1.80
3	4.5	33.0		2.33	1.80	2.54	1.62
4	7.9	94.4		1.66	1.93	2.98	2.00
Median	7.1	100.2	0.0209	1.49	1.80	1.58	1.80

The mean concentrations of Group 1 (63.21 ± 56.68 ng/μL), Group 2 (52.58 ± 71.42 ng/μL) and Group 3 (63.76 ± 57.35 ng/μL) were significantly higher than those of Groups 4 (2.11 ± 3.38 ng/μL) and 5 (4.35 ± 6.01 ng/μL; F = 4.430; *p* = 0.004). The mean 260/280 ratio values were similar among the five groups (F = 0.363; *p* = 0.834); however, 81.8% of Groups 1, 2 and 3, 9.1% of Group 4 and 50.0% of Group 5 had a260/280 ratio between 1.8 and 2.0 (*p* = 0.003). The mean 260/230 ratio values of Group 1 (1.24 ± 0.93 nm), Group 2 (1.35 ± 0.60 nm) and Group 3 (1.26 ± 0.70 nm) were significantly higher than those of Group 4 (0.07 ± 0.10 nm) and Group 5 (0.25 ± 0.37 nm; F = 10.904; *p* <  0.001). Moreover, 45.5% of Groups 1 and 2, 27.3% of Group 3, and 0% of Groups 4 and 5 had a 260/230 ratio between 1.8 and 2.2 (*p* = 0.001, Table 2, Fig. 2d-f).

**Table 2:** Comparison of RNA quantity and quality among the five groups

Group	Concentration (ng/ μL)	Statistics, *P*	A260/280 (nm)	Statistics, *P*	A260/230 (nm)	Statistics, *P*	GAPDH density	Statistics, *P*	E6 density	Statistics, *P*
1 ^a^	63.21 ± 56.68^d,e^	F = 4.430, 0.004	2.23 ± 0.66	F = 0.363, 0.834	1.24 ± 0.93^d,e^	F = 10.904,< 0.001	58,736.45 ± 21,574.01^d,e^	F = 17.682,< 0.001	37,511.94 ± 33,672.71^c,d,e^	F = 4.102, 0.011
2^b^	52.58 ± 71.42^d,e^		2.14 ± 0.49		1.35 ± 0.60^d,e^		58,456.82 ± 14,483.19^d,e^		17,286.74 ± 18,989.13^c,d,e^	
3^c^	63.76 ± 57.35^d,e^		2.56 ± 2.17		1.26 ± 0.70^d,e^		50,556.82 ± 27,070.25^d,e^		9593.98 ± 8535.84^a,b^	
4^d^	2.11 ± 3.38^a,b,c^		2.73 ± 3.38		0.07 ± 0.10^a,b,c^		12,229.28 ± 8516.42^a,b,c^		2273.92 ± 1423.42^a,b^	
5^e^	4.35 ± 6.01^a,b,c^		1.85 ± 0.78		0.25 ± 0.37^a,b,c^		8633.20 ± 20,804.91^a,b,c^		2157.26 ± 2037.34^a,b^	

Data are presented as means ± standard deviations

The sample letters (a, b, c, d, and e) indicate non-significant differences between groups

*P-*values were assessed by ANOVA and post hoc Student-Newmann-Keuls test

**Fig. 3:**
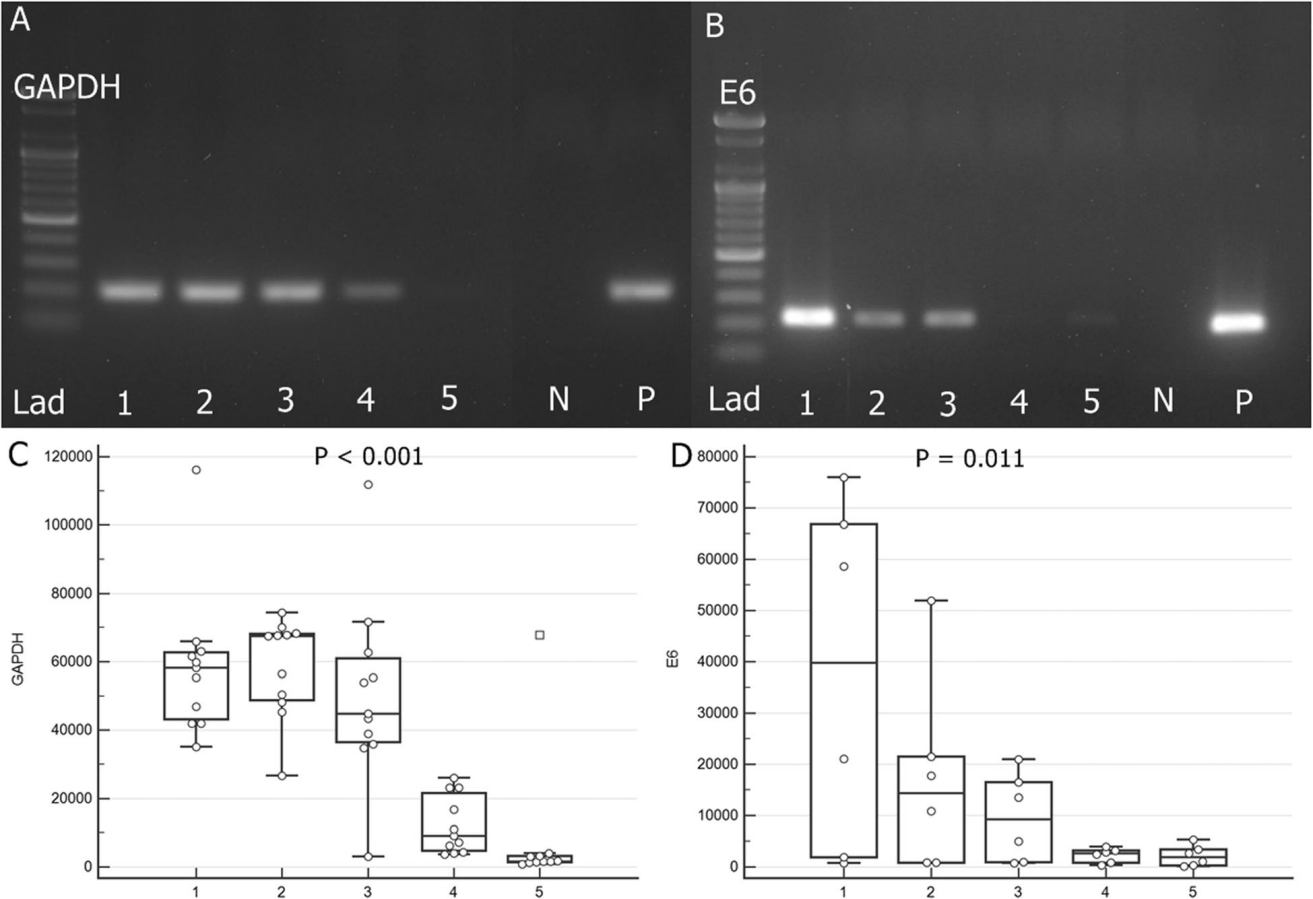
Agarose gel electrophoresis for GAPDH (**a**) and E6 (**b**). Comparisons densities of GAPDH (**c**) and E6 (**d**) among the five groups. Lad = 100 bp DNA Ladder (molecular weight marker); N = negative control; P = positive control; the same gels were used

The mean GAPDH densities of Group 1 (58,736.45 ± 21,574.01), Group 2 (58,456.82 ± 14,483.19), and Group 3 (50,556.82 ± 14,483.19) were significantly higher than those of Group 4 (12,229.23 ± 8516.42) and Group 5 (8633.20 ± 20,804.91; F = 17.682; *p* <  0.001). The mean E6 densities of Group 1 (37,511.94 ± 33,671.71) and Group 2 (17,286.74 ± 18,989.13) were significantly higher than those of Group 3 (9593.98 ± 8535.84), Group 4 (2273.92 ± 1423.42) and Group 5 (2157.26 ± 2037.34; F = 4.102; *p* = 0.011, Table 2, Fig. 3).

This study provides a comparative analysis of the mRNA quantity and quality of cervico-vaginal cytology samples as affected by tube type, storage temperature, and preservative solution. Soft tubes enhanced the mRNA quantity and quality compared to the conventional rigid tubes usually used in cervico-vaginal cytology. We developed a novel method to separate exfoliated cells from cervico-vaginal smears from the brush using soft, elastic tubes. We could separate the exfoliated cells from the brush by squeezing the soft tube. The quantity and quality of the mRNA extracted using the soft tube was excellent compared to that obtained from the conventional rigid tubes in this study. The productivities of GAPDH mRNA extraction using RNAlater RNA Stabilization Reagent and RNAprotect Cell Reagent, and that of HPV 16 E6 using RNAlater RNA Stabilization Reagent at room temperature were comparable to those of conventional frozen samples.

RNA samples obtained by cytology are partially degraded, leading to low levels of transcript detection [9]. RNases which enzymatically degrade RNA are nearly ubiquitous and pose a constant threat of contamination and degradation of purified RNA. Homogenate RNase exists at high levels in the vaginal fluid of cervical cancer patients [10]. Frozen specimens are traditionally used for translational research and contain well-preserved nucleic acids and proteins. However, equipment, space, power, maintenance, and handling costs for frozen specimens are substantial over time. Freezer failure is a real concern, as demonstrated by loss of one third of the specimens in a national autism brain bank [11]. Many commercial RNA stabilization reagents are therefore available for producing purified RNA which can be stored at room temperature. In our study, the RNA quantity and quality produced using two RNA stabilization reagents, RNAlater RNA Stabilization Reagent and RNAprotect cell Reagent, were comparable to those achieved by frozen storage. However, AllProtect Tissue Reagent showed inferior RNA quantity and quality compared to frozen storage. AllProtect Tissue Reagent contains the stickiest solution among the three RNA stabilization reagents, and this may affect the separation of the exfoliated cells from the brush. Most cervico-vaginal cytology samples were acquired from patients during medical treatment. Under these conditions immediate delivery to the freezer may difficult, and room-temperature tissue storage with stabilizing reagents is important. Although RNA stabilization reagents are not a long-term solution (7 days at room temperature), the ability to store and freeze tissue in stabilization reagents may be valuable for gaining time until delivery to the freezer and may provide insurance against unexpected freezer failure or temperature fluctuations. Moreover, our novel method may contribute to molecular studies using other exfoliated cells, such as oral cytology.

Previous studies have demonstrated that RNA can be extracted from cervical cell lines and cytology specimens can be stored in BD Surepath™ preservative fluid, and the RNA is suitable for RT PCR [12]. In our study, the quantity and quality of RNA using BD Surepath™ preservative fluid was inferior to that of conventional frozen storage. Powell et al. reported that storage of cells in Surepath resulted in significantly reduced yields [13]. Reduced recovery of RNA could result from exposure to components of the preservative fluid which could result in chemical modification of the RNA, such that it is no longer an amenable substrate for the reverse transcriptase.

To the best of our knowledge, this is the first study into enhancing RNA extraction methods for cervico-vaginal cytology samples according to tube types, storage temperatures, and preservative solutions. By squeezing the soft tube and brush, we could acquire more cells from the brushes, and hence could enhance the quality and quantity of RNA extracted, compared to conventional rigid tubes. Moreover, room temperature storage in RNAlater RNA Stabilization Reagent and RNAprotect cell Reagent was comparable to conventional frozen storage in terms of RNA quantity and quality. Our improved RNA extraction method may enhance the quantity and quality of RNA cervico-vaginal cytology samples and contribute to successful molecular studies using cervico-vaginal cells and other exfoliated cell.

## Supplementary information

**Additional file 1: Table S1.** Primer sets used for Polymerase Chain Reaction of GAPDH and E6 genes.

## Data Availability

Not applicable.

## Abbreviations

DNA: Deoxyribonucleic acid
GAPDH: Glyceraldehyde 3-phosphate dehydrogenase
HPV: Human papillomavirus
mRNA: messenger Riboucleic Acid
RNase: Ribonuclease
RT PCR: Reverse transcription polymerase chain reaction
SPSS: Statistical product and service solutions
