# Supplementary material for: Improvement in RNA quantity and quality in cervico-vaginal cytology
Source: Virol J. 2020 Jan 20;17:8. doi: 10.1186/s12985-020-1282-x (PMC6971917; doi:10.1186/s12985-020-1282-x)
Supplement: Supplementary file 1 — Additional file 1: Table S1. Primer sets used for Polymerase Chain Reaction of GAPDH and E6 genes. [file 12985_2020_1282_MOESM1_ESM.docx]

Supplementary Table 1. Primer sets used for Polymerase Chain Reaction of GAPDH and E6 genes.

| Primer | Sequence (5’-3’) | Products Size of mRNA |
| --- | --- | --- |
| GAPDH F | GGA GCG AGA TCC CTC CAA AAT | 197 bp |
| GAPDH R | GGC TGT TGT CAT ACT TCT CAT GG |  |
| E6 F | ACT GCG ACG TGA GGT ATA TGA CT | 225 bp |
| E6 R | GGA CAC AGT GGC TTT TGA CAG TT |  |
